# RNA-Seq Profiling of Serum Exosomal Circular RNAs Reveals Circ-PNN as a Potential Biomarker for Human Colorectal Cancer

**DOI:** 10.3389/fonc.2020.00982

**Published:** 2020-06-18

**Authors:** Yan Xie, Juan Li, Peilong Li, Ning Li, Ying Zhang, Helen Binang, Yinghui Zhao, Weili Duan, Yingjie Chen, Yunshan Wang, Lutao Du, Chuanxin Wang

**Affiliations:** ^1^Department of Clinical Laboratory, The Second Hospital of Shandong University, Jinan, China; ^2^Tumor Marker Detection Engineering Laboratory of Shandong Province, Jinan, China

**Keywords:** colorectal cancer, serum exosomes, circRNA, biomarkers, diagnosis

## Abstract

Circular RNAs (circRNAs) are emerging as cardinal areas of focus in the non-coding RNA field. Growing evidences have revealed exosomal circRNAs as potential biomarkers for detection of various cancers. However, the clinical importance of most serum exosomal circRNAs in colorectal cancer (CRC) have rarely been investigated. In this study, we examined the possible clinical application of serum exosomal circRNAs in the diagnosis of CRC. Firstly, we conducted RNA sequencing (RNA-seq) analysis using fifty CRC and fifty healthy control serum samples to identify CRC-related circRNAs. The sequencing data showed 122 differentially expressed circRNAs including 100 up-regulated and 22 down-regulated circRNA transcripts in CRC patients. Then, eight most dysregulated circRNAs were selected for validation by reverse transcription-quantitative polymerase chain reaction (RT-qPCR) assay. Validation analysis revealed that the serum exosomal circ-PNN (hsa_circ_0101802) levels were significantly up-regulated in CRC cases compared with those in the healthy control groups. Receiver operating characteristic curve (ROC) analysis suggested that circ-PNN had significant value in CRC diagnosis with areas under the ROC curve (AUC) of 0.855 and 0.826 in the training and validation sets, respectively. We also found that the AUC of serum exosomal circ-PNN for early-stage CRC was 0.854. Finally, a network map based on circ-PNN was constructed to determine its potential miRNA-mRNAs binding. We also demonstrated that the expression of hsa-miR-6833-3P, hsa-let-7i-3p and hsa-miR-1301-3P were negatively correlated with circ-PNN in CRC patients. Collectively, our findings indicated that serum exosomal circ-PNN might be a potential non-invasive biomarker for the detection of CRC and may play a crucial role in the pathogenesis of CRC.

## Introduction

Colorectal cancer (CRC) is the third most common malignant cancer in both men and women, and ranks the second leading cause of cancer deaths in the world. It is estimated that over 1.8 million new CRC cases and 881,000 deaths occurred in 2018, accounting for approximately one in ten cancer cases and deaths (1). Despite many advances achieved in diagnosis and therapy, the prognosis of CRC patients remains unsatisfactory due to the advanced stage at first diagnosis and the high frequency of metastasis and recurrence (2). Statistics from well-known sources have reported that the 5-year survival rates for CRC patients with early-stage disease was 90%, and the survival rate was only 13.1% for those diagnosed with late-stage CRC (3, 4). Therefore, to raise the efficiency of tumor diagnosis and improve the prognosis of CRC patients, new sensitive and cost-effective biomarkers are urgently required.

Exosomes are 50–150 nm disk-shaped microvesicles, which are released from multiple cells into extracellular space (5). Previous reports have shown that exosomes are commonly found in nearly all body fluids including blood, urine, saliva, and ascites. Their stability in adverse environments have also been confirmed in many body fluids (6, 7). Extracellular vesicles, including tumor-derived exosomes, contain substantial amounts of proteins, DNA, and RNA that reflect the originating tumor cells (8–10). Many studies have suggested possible utilization of exosomal RNAs as reliable indicators in the diagnosis and prognosis of various cancers (11–13).

Circular RNAs (circRNAs) represent a new class of endogenous non-coding RNAs, characterized by covalently closed cyclic structures and lacking 5′-3′ ends and poly A tail (14–17). Owing to this structure, circRNAs are free from RNase R and exhibit higher stability than linear RNAs, which endows them with many potential functions and applications (15). The cell type or developmental stage-specific expression patterns have made them new molecular markers for cancer diagnosis (16). However, the expression profiles of newly identified circRNAs, and the possibility of utilizing serum exosomal circRNAs as biomarkers in CRC still require further investigation.

In our study, we analyzed the expression profile of serum exosomal circRNAs in CRC patients to find potential circRNA biomarkers, and identified circ-PNN (hsa_circ_0101802) significantly upregulated and closely related with the prognosis of CRC patients. We also explored the potential clinical significance of serum exosomal circ-PNN and unveiled that circ-PNN level had good performance to distinguish CRC cases from healthy controls. Therefore, circ-PNN may serve as a diagnostic biomarker for CRC.

## Results

### CircRNAs Expression Profiles

**Figure 1:**
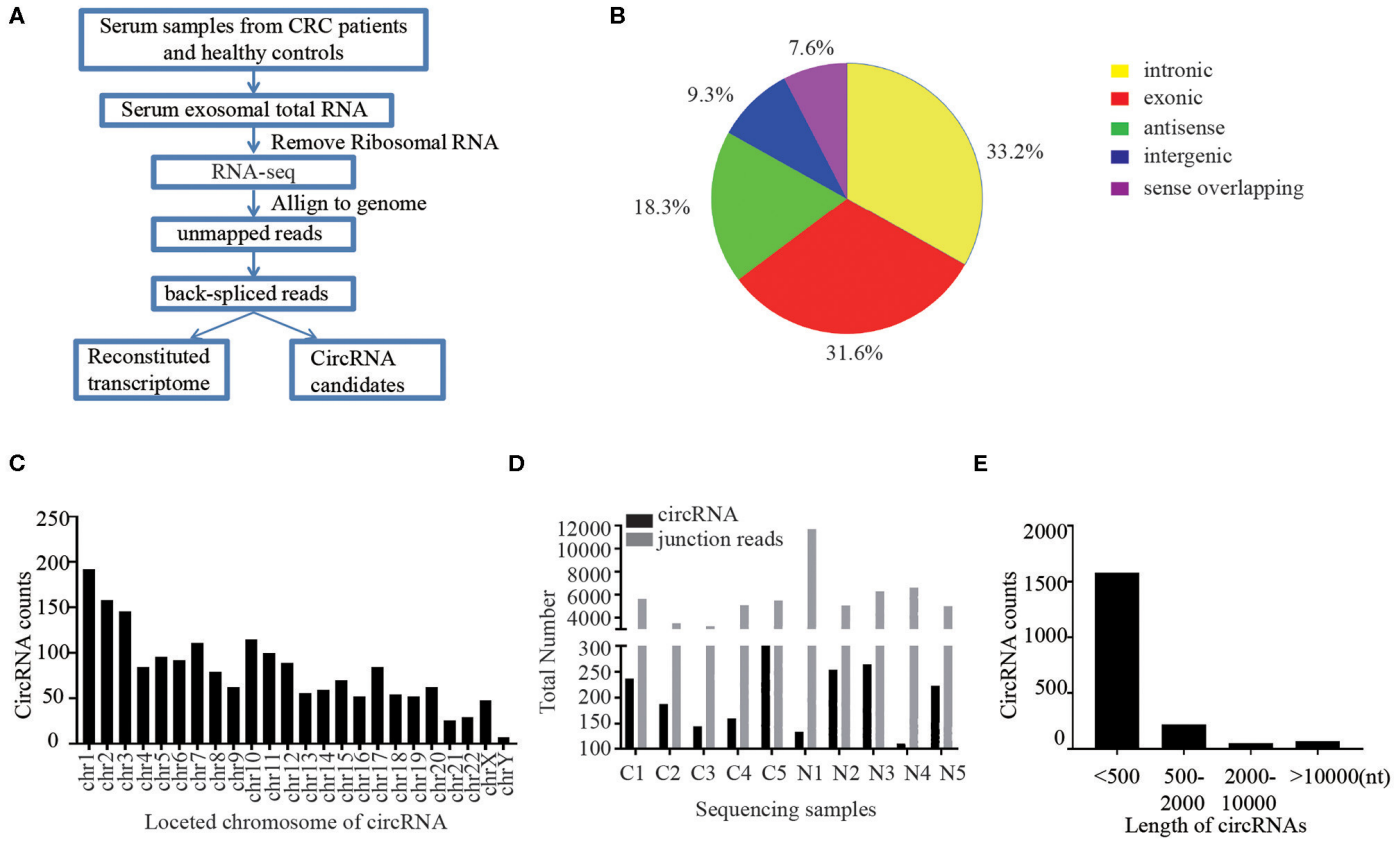
Profiling of circular RNAs in human serum exosomes. **(A)** Workflow for RNA-seq analysis of serum exosomal circRNAs from CRC patients and healthy controls. **(B)** Classification of identified circular RNAs based on the genomic origin. **(C)** The distributions of identified circRNAs in different chromosomes. **(D)** The number of circular RNAs and back spliced reads detected in each sample. **(E)** Length distribution of the identified circRNAs.

First of all, we performed RNA sequencing (RNA-seq) analysis of ribosomal RNA-depleted total RNA from 50 CRC patients and 50 healthy controls to generate a circRNA profiling database. The basic characteristics of the CRC patients and healthy controls are summarized in Table S1. RNA-seq reads were then aligned to the reference genome/transcriptome, the ones contiguously mapped were discarded, and the remaining reads were used to identify circular RNAs by analyzing the head-to-tail splice junction via DCC software (Figure 1A). A total of 1,924 circRNAs were identified based on at least one read spanning a back-splice junction.

Due to the different cycling mechanisms of circRNAs, they can be divided into five categories according to their locational relationship with adjacent coding RNAs, including exonic circRNAs, intronic circRNAs, intergenic circRNAs, antisense circRNAs and sense overlapping circRNAs (18). Interestingly, among the 1,924 identified circRNAs, 33.2% were intronic circRNAs, 31.6% were exonic circRNAs, 18.3% were antisense circRNAs, and 16.9% of the circRNAs were of other sources (Figure 1B). We further sorted these identified circRNAs base on their different locations on chromosomes. The results showed that these circRNAs were widely distributed across all chromosomes (Figure 1C). Chromosomes 1–20 contained more than 50 circRNAs each and chromosome X had 48 circRNAs, while the chromosomes 21–22 had ~ 30 circRNAs and Y chromosomes contained <10 circRNAs. The total number of circular RNAs and junction reads detected in different serum exosome samples are shown in Figure 1D. Majority of the identified circRNAs were <2,000 nucleotides (nt) in length, and most were <500 nt long (Figure 1E).

### Different CircRNAs Expression Patterns in CRC Patients and Healthy Controls

**Figure 2:**
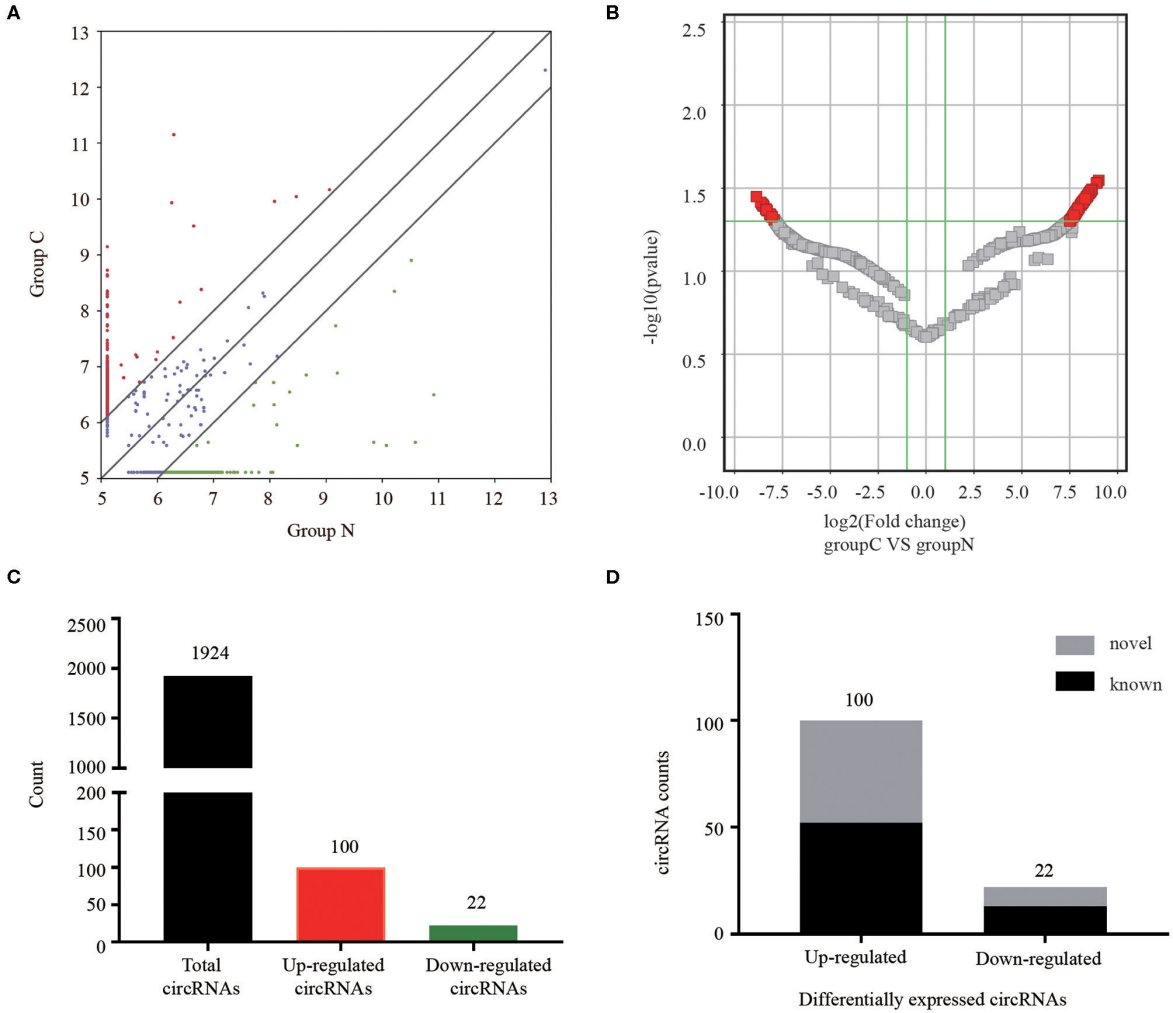
Analysis of circRNAs in CRC patients and healthy controls by RNA-sequencing. **(A)** The scatter plot revealed different circRNA expression profiles between CRC patients (C) and healthy controls (N). Red points indicated upregulated circRNAs with FC≥2.0 in CRC patients, and green points represented downregulated circRNAs. **(B)** Volcano plot showing differential expressions of circRNAs between the two groups. The vertical green lines depict a 2.0-fold (log2 scaled) up or down changes, while the horizontal green line marks a *P*-value of 0.05 (–log10 scaled). Red points in the plot represent significantly differentially expressed circRNAs. **(C)** The amount of the total identified circRNAs and differentially expressed circRNAs. **(D)** Among the circRNAs with statistically significant differences in expression, gray indicated novel circRNAs, and black represented known circRNAs.

The serum exosomal circRNAs differentially expressed between CRC patients and healthy control groups were determined according to a statistical criteria of fold change ≥2.0 and *P-*value ≤ 0.05. We found 122 circular RNAs whose expressions were significantly different. A scatter plot (Figure 2A) and a volcano plot (Figure 2B) of all the differentially expressed circRNAs were generated to present the distinguishable circRNA expression profiles. Among the 1,924 identified circRNAs, 100 were significantly up-regulated and 22 were significantly down-regulated (Figure 2C). Among the 122 differentially expressed circRNAs, 65 were confirmed as novel circRNAs, including 52 up-regulated and 13 down-regulated circRNAs, and the remaining identified circRNAs are listed in the circBase or article (Figure 2D).

### Predicted Functions of Differentially Expressed CircRNAs in CRC Patients

**Figure 3:**
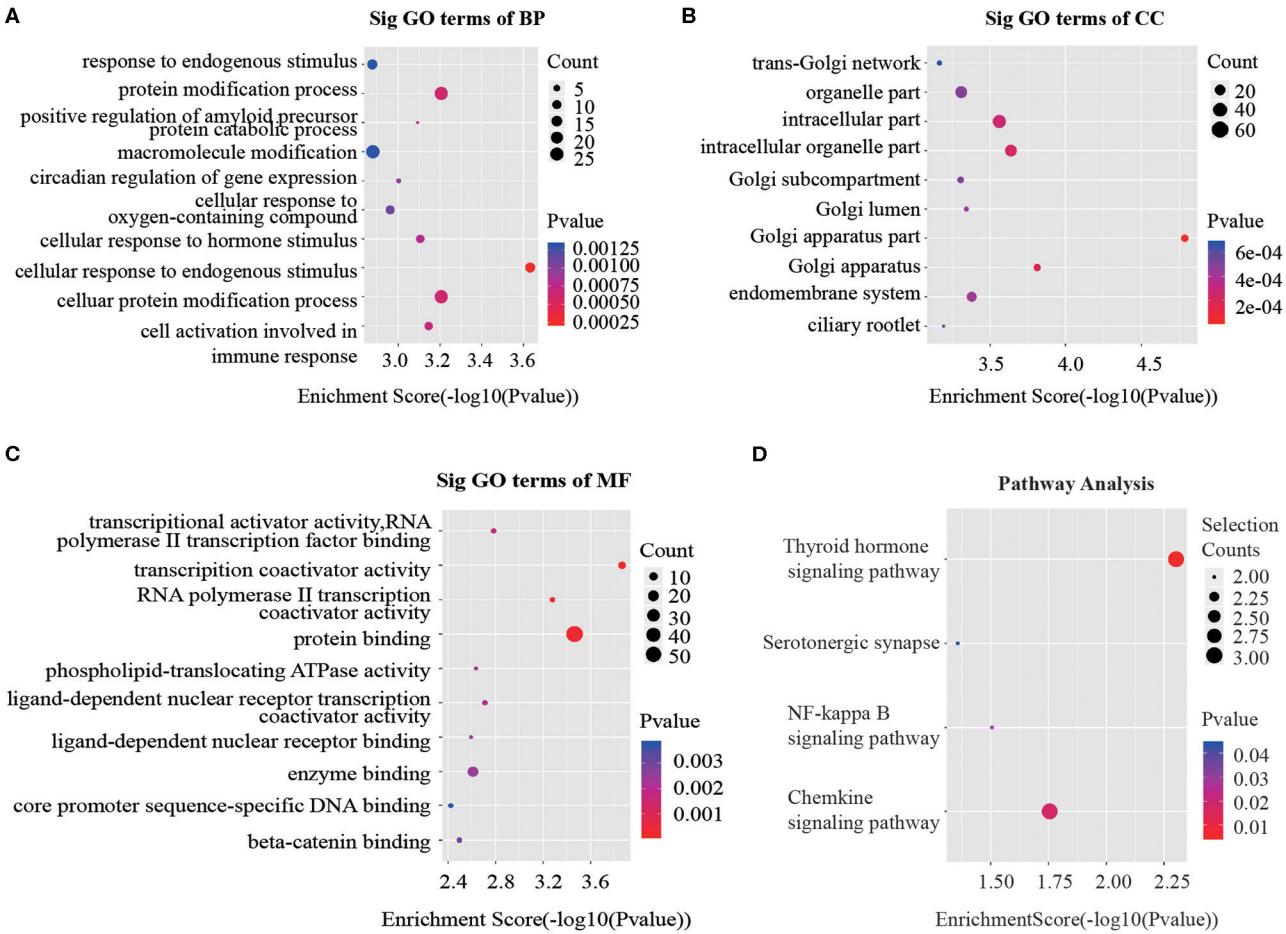
GO and KEGG signaling pathway analysis of the parent gene regulated by overexpressed circRNAs in serum exosomes. Gene ontology analysis consists of **(A)** biological processes, **(B)** cellular components and **(C)** molecular functions. **(D)** Top 10 significantly enriched pathway terms related to the up regulated circRNAs. The horizontal axis is the –Log *P* (logarithm of *P*-value) for the enriched GO terms (pathway) and the vertical axis is the GO terms (pathway) category. *P* < 0.05 was considered significant.

Recent studies have demonstrated that circRNAs may regulate the expression of the parental genes they are derived from (19, 20). According to the contributions of parental genes to biological processes, cellular components and molecular functions and pathways, we performed Gene Ontology (GO) and Kyoto Encyclopedia of Genes and Genomes (KEGG) analysis for circRNAs to predict their potential functions. Considering that majority of the differentially expressed circRNAs were up-regulated, we focused on GO analysis of the 100 noteworthy up-regulated circRNAs. The lower the *P*-value, the more significant the correlation. Three significantly enriched GO terms in biological processes identified were “cellular response to endogenous stimulus,” “cellular protein modification process” and “protein modification process” (Figure 3A). When classified according to cellular component, three most significantly enriched GO terms were “Golgi apparatus part,” “Golgi apparatus” and “intracellular organelle part” (Figure 3B). Based on molecular functions, three significantly enriched GO terms were “protein binding,” “transcription coactivator activity,” and “RNA polymerase II transcription coactivator activity” (Figure 3C).

The KEGG analysis made it possible to speculate biological functions of significantly up-regulated circRNAs through pinpointing pathways relevant to their host genes. We found 4 signaling pathways that were possibly affected by CRC as shown in Figure 3D.

### Characterization of Serum Exosomes

**Figure 4:**
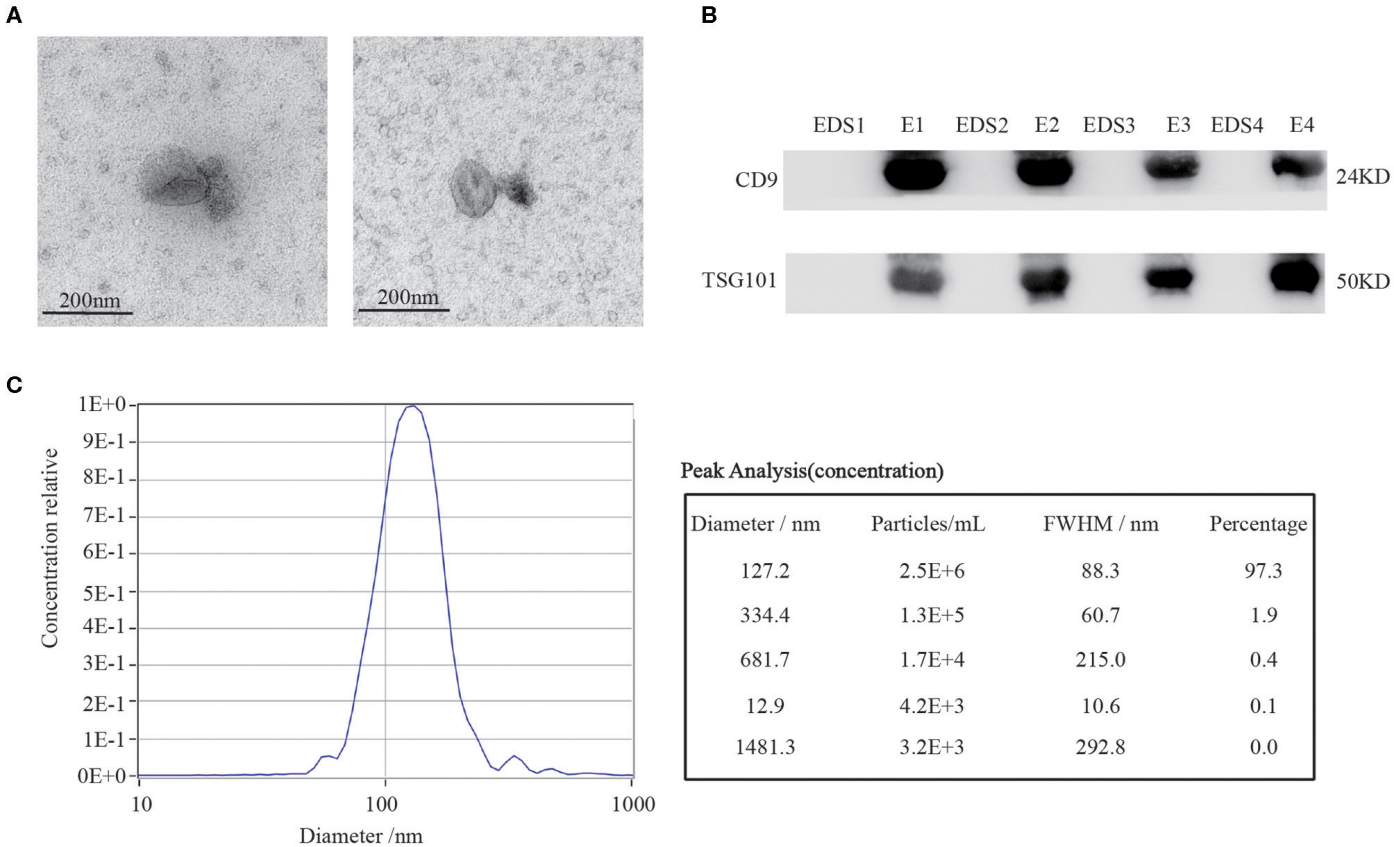
Characterization of serum exosomes. **(A)** TEM images confirmed the presence of exosomes. Scale bar=200 nm. **(B)** Exosomes-enriched protein markers including CD9 and TSG101 were analyzed in exosomes (E) and exosome-depleted supernatant (EDS) by Western blot analysis. **(C)** The sizes of serum exosomes were determined via the NTA characterization system.

To study the expression profiles of our target circRNAs in CRC serum exosomes, we initially isolated and purified the exosomes as follows: Firstly, the supernatant of venous blood was collected, and the exosomes were extracted using commercial kits and identified. To confirm that the pellets obtained via the exosomes precipitation protocol were truly exosomes, transmission electron microscopy (TEM) was performed to show the typical oval-shaped extracellular vesicles with diameters of ~ 50–150 nm (Figure 4A). Western blot analysis demonstrated that CD9 and TSG101 were readily detected in the exosomes but not in the exosome-depleted supernatant (EDS) (Figure 4B). Meanwhile, this finding was further confirmed by nanoparticle tracking analysis (NTA) (Figure 4C). Together, these results showed that exosomes had indeed been adequately purified from serum, which laid a foundation for further study of serum exosomal biomarkers.

### The Biological Structure of Circ-PNN

Considering that down-regulated circRNAs are not readily associated with disease progression because of their low expression levels, we selected 8 up-regulated circRNAs which had the most significantly different expressions (Table S2). We then designed divergent primers for those circRNAs, and the melting curve confirmed the amplified products by a single peak. PCR produced a band of the expected size, and we gel-extracted and sequenced those products, confirming that they contained the head-to-tail junction. For “chr20:50245424- 50245575-”, we tried different primers to amplify this circRNA, but all of these primers caused nonspecific amplification.

**Figure 5:**
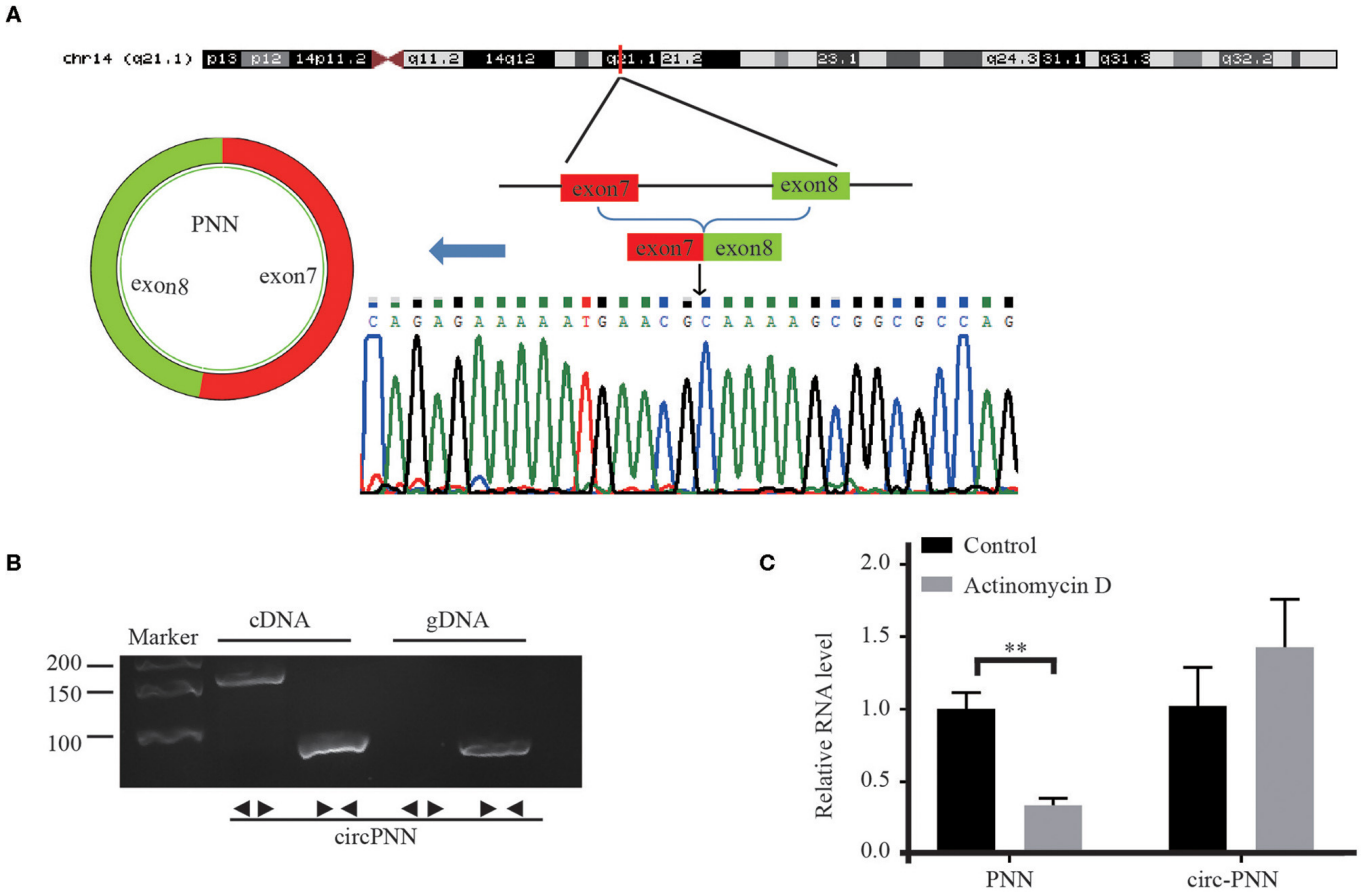
Characterization of Circ-PNN. **(A)** Genomic loci of circ-PNN gene, determined by Sanger sequencing following RT-qPCR, confirmed the “head-to-tail” splicing of circ-PNN. **(B)** PCR analysis showed that circ-PNN was only amplified by divergent primers in reverse-transcribed RNA (cDNA), but not in genomic DNA (gDNA). **(C)** RT-qPCR detection of PNN and circ-PNN expression in SW620 cells with or without 2 mg/mL Actinomycin D treatment for 12 h. **P < 0.01.

We assessed the structure of circ-PNN, which is derived from exon 7 to exon 8 of PNN gene. Subsequently, we confirmed the back-spliced junction in the PCR product of circ-PNN with expected size by Sanger sequencing (Figure 5A). The sequence was consistent with circBase database annotation. In addition, PCR analysis for reverse-transcribed RNA (cDNA) and genomic DNA (gDNA) showed that circ-PNN was only amplified by divergent primers in cDNA, but not in gDNA (Figure 5B). Furthermore, to determine the stability of circ-PNN, total RNA was treated with Actinomycin D, an inhibitor of transcription. After treatment with Actinomycin D, reverse transcription-quantitative polymerase chain reaction (RT-qPCR) analysis showed that circ-PNN transcripts were stable in comparison to PNN mRNA, indicating that circ-PNN is more stable in CRC cells (Figure 5C). Altogether, these analyses demonstrated that circ-PNN has a bona fide circRNA structure.

### Selection and Evaluation of Candidate Serum Exosomal CircRNAs in CRC Patients

**Figure 6:**
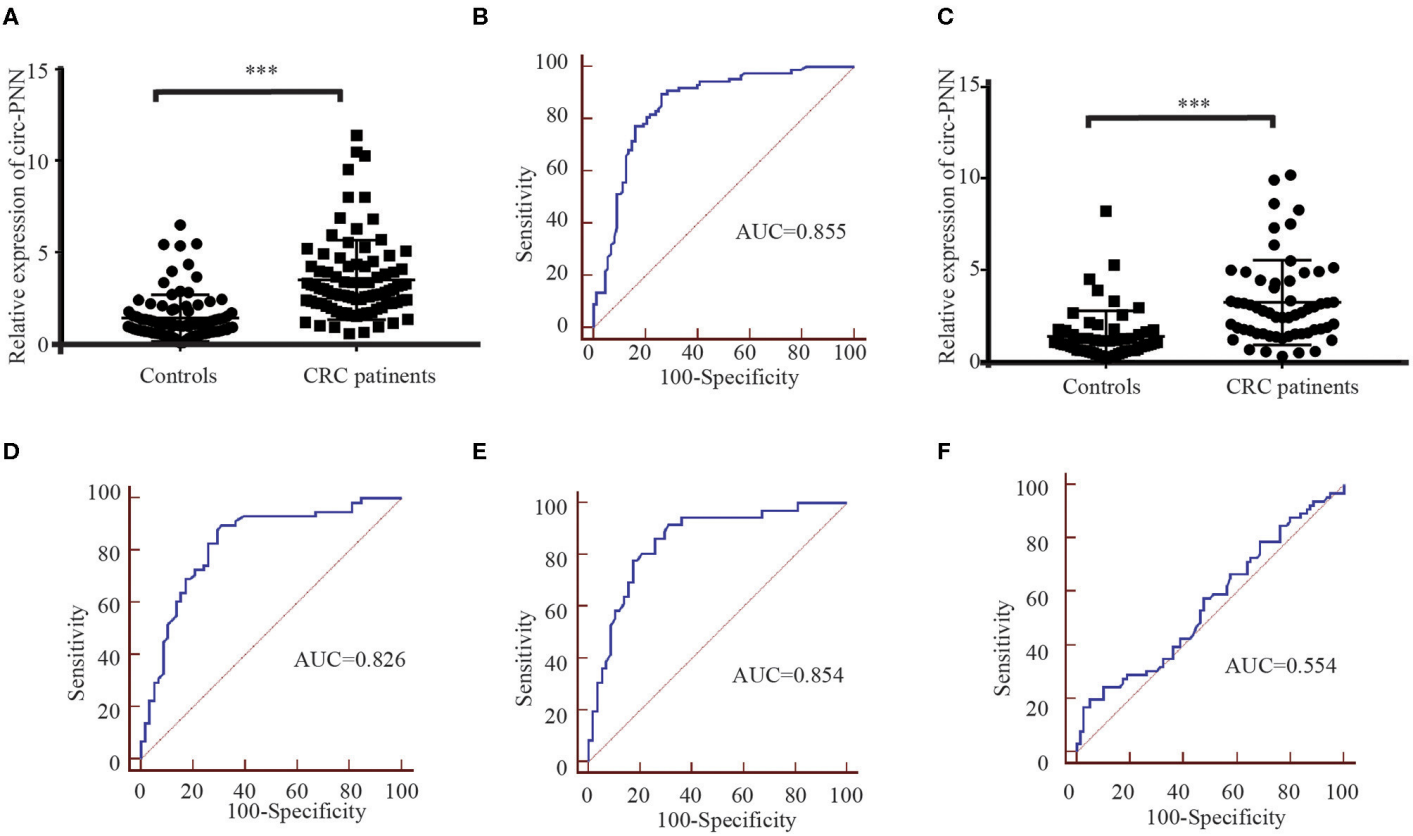
Correlation between the circ-PNN and CRC. **(A)** The relative expression of serum exosomal circ-PNN (relative to β-actin) in patients with CRC (*n* = 88) and healthy controls (*n* = 88) determined using RT-qPCR assay in the training set, ***P < 0.001. **(B)** ROC curve analysis for the determination of the diagnostic performance of circ-PNN in the training set. **(C)** The relative expression of serum exosomal circ-PNN (relative to β-actin) in patients with CRC (*n* = 58) and control individuals (*n* = 58) determined using RT-qPCR assay in the validation set, ***P < 0.001. **(D)** ROC curves for the determination of the diagnostic performance of circ-PNN in the validation set. **(E)** ROC curve showing the capability of serum exosomal circ-PNN in distinguishing patients with early-stage CRC from normal healthy individuals. **(F)** ROC curve showing the capability of serum exosomal circ-PNN in distinguishing patients with Lymph node metastasis.

In the training set, we performed RT-qPCR using serum exosome samples derived from 88 CRC patients and 88 healthy controls to verify the circRNA-seq results. Among the selected circRNAs, the expression levels of circ-PNN (*P* < 0.001) in CRC patients were apparently higher than in healthy controls (Figure 6A), which was consistent with previous sequencing results. The potential diagnostic values of significantly differentially expressed circRNAs were evaluated through Receiver operating characteristic curve (ROC) analysis. The area under the ROC curve (AUC) of serum exosomal circ-PNN in training set was 0.855 (95% CI = 0.794–0.904, sensitivity = 89.8% and specificity =73.9%, Figure 6B).

Subsequently, the expression of circ-PNN was further verified in the validation set (58 CRC patients and 58 healthy controls). The dysregulated expression trend was consistent with the training set (Figure 6C), and the diagnostic accuracy of circ-PNN in the validation set, measured by AUC, was 0.826 (95% CI = 0.745–0.890, sensitivity = 89.7% and specificity = 69.0%, Figure 6D). These data indicate that abnormal circ-PNN expression may be related to CRC progression. Demographic and clinical features of the CRC patients and controls in traning set and validation set are summarized in Table S3.

### Correlation Between Circ-PNN and Clinicopathological Characteristics

Finally, to determine whether the expression levels of serum exosomal circ-PNN was related to the progress of CRC, we analyzed the correlation between the serum exosomal circ-PNN and clinicopathological characteristics of the CRC patients. However, no significant associations were found between the exosomal circ-PNN and clinicopathological characteristics (Table S4). In our study, we considered CRC patients at TNM stage I/II as early CRC patients. And the potential diagnostic values of exosomal circ-PNN for early-stage CRC and status of lymph node metastasis (LNM) in the validation set were evaluated through ROC curve analysis. The AUC of serum exosomal circ-PNN for early-stage CRC was 0.854 (95% CI = 0.766 to 0.918, sensitivity = 91.7% and specificity = 69.0%) (Figure 6E). However, the AUC of serum exosomal circ-PNN for LNM status was only 0.554 as shown in Figure 6F. Therefore, circ-PNN may serve as a diagnostic biomarker for early-stage CRC.

### CircRNA-miRNA-mRNA Network Prediction and Analyses

**Figure 7:**
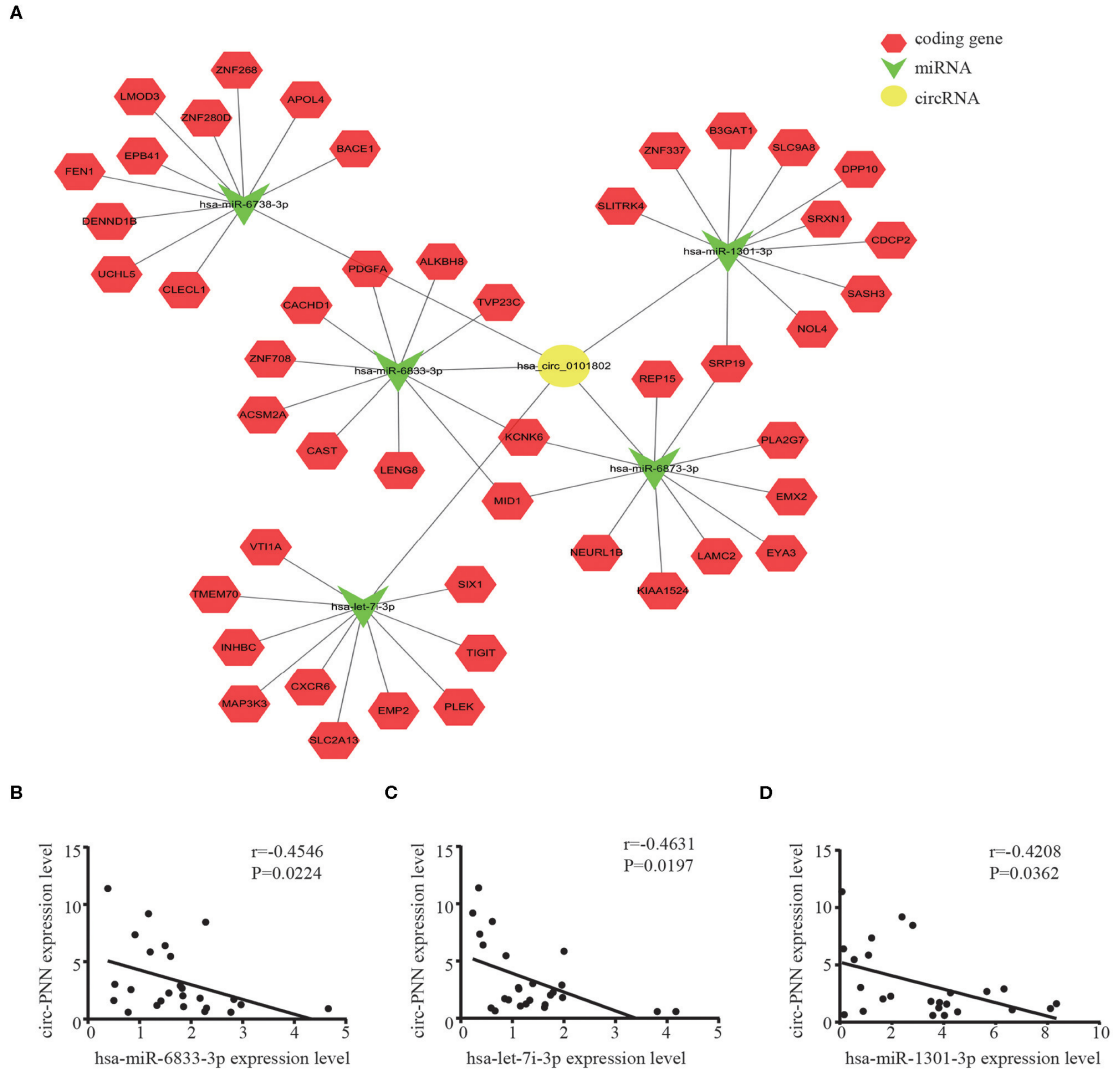
CircRNA-miRNA-mRNA network prediction and analyses. **(A)** The possible binding of miRNAs and mRNAs to circ-PNN. Circles represent circRNAs, arrowheads represent miRNAs, hexagons represent mRNAs, red represents coding gene. The expression of hsa-miR-6833-3P **(B)** hsa-let-7i-3p **(C)** and hsa-miR-1301-3P **(D)** all showed negative correlation with levels of circ-PNN in CRC.

We then built a circRNA-miRNA-target gene network for circ-PNN using Cytoscape. The top 5 miRNAs that potentially bind to circ-PNN and the five most likely target genes to each miRNA were used to construct a network map (Figure 7A). The network gave us a clear direction for future study of the underlying mechanism of circ-PNN in the tumorigenesis or development of CRC. We conducted a preliminary verification of the results. A total of 25 serum samples of CRC patients, and 25 serum samples of healthy controls were obtained to analyze the expression correlation between circ-PNN and miRNAs, and the personal characteristic of participants were shown in Table S5. Regarding the top 5 miRNAs, we tried different primers to amplify hsa-miR-6738-3p, but all of these primers caused non-specific amplification, and the expression levels of hsa-miR-6873-3p was too low to be detected. Therefore, we investigated the interaction of circ-PNN with the remaining three miRNAs. Consistent with our prediction, we found that the expression of hsa-miR-6833-3p (Spearman's correlation, r = −0.4546, *P* < 0.05), hsa-let-7i-3p (Spearman's correlation, r = −0.4631, *P* < 0.05) and hsa-miR-1301-3p (Spearman's correlation, r = −0.4208, *P* < 0.05) showed negative correlation with levels of circ-PNN in CRC (Figures 7B–D).

## Discussion

The initiation and development of CRC in the colonic epithelium is closely associated with heredity and several environmental factors (21). The detection of early CRC has become a huge challenge since patients with advanced CRC usually have a poor prognosis (22, 23). Although significant progress has been made in the diagnosis of CRC in recent decades, such as imaging and hematological examinations, they were limited due to their low sensitivity and specificity. Therefore, discovery of effective biomarkers remains imperative to raise the efficiency of CRC diagnosis. CircRNAs are a new type of non-coding RNAs, which are abundant and stable in a variety of body fluids than linear RNAs and may serve as new reliable diagnostic biomarkers for cancers (24–26). In the present study, we analyzed the relevance of serum exosomal circRNAs in CRC compared to the healthy control group according to the results of circRNAs sequencing, and then verified circ-PNN which we chose for further ROC curve analysis. We interestingly found that circ-PNN has high diagnostic efficacy for early CRC. Subsequently, the potential miRNA targets of circ-PNN were predicted by miRNA target predication software based on sequence pairing. In addition, circ-PNN displayed a tendency of negative correlation with hsa-miR-6833-3p, hsa-let-7i-3p and hsa-miR-1301-3p in CRC.

Recently, circRNAs have attracted significant attention as ideal diagnostic markers for cancers than miRNAs and lncRNAs for certain reasons. First, circRNAs are characterized by stable structures which are resistant to RNase activity and higher cell and tissue specificity than liner RNAs (27, 28). Secondly, Genome-wide analyses of RNA sequencing data have showed evolutionary conservation and abundance of circular RNAs (29, 30); and thirdly, they are plentiful in various body fluids including blood, saliva, serum, and even in exosomes. These advantages make circRNAs potential candidates as liquid biopsy biomarkers (24, 31–33). Previous research reported that circRNAs have the function of promoting CRC cells growth and metastasis. For example, circular RNAs ciRS-7 and hsa_circ_0000069 have been found to be significantly over-expressed in CRC tissues and might serve as potential novel and stable biomarkers for the diagnosis of CRC (34, 35). Zhu et al. (36) also screened the differentially expressed circRNAs by using a circRNA microarray approach. The most significantly up-regulated circRNA, hsa_circ_0007142, was chosen for further study and they found that hsa_circ_0007142 was associated with the lymphatic metastasis and differentiation of CRC (36). A recent study also demonstrated the existence of abundant exo-circRNAs in the serum of CRC patients, which were suggested as potential circulating biomarkers for cancer diagnosis (24). However, that study was limited by the small sample sizes, and the lack of independent validation. As far as we know, this is the first report that exhaustively investigates the expression and clinical implication of serum exosomal circ-PNN in CRC. In this study, we analyzed the clinical revelance of serum exosomal circRNAs in CRC patients compared with healthy controls by circRNAs sequencing. After comprehensive consideration, we performed ROC curve analysis to verify the diagnostic efficacy of serum exosomal circ-PNN from two independent sample sets (including training and validation sets). And the AUC were 0.855 and 0.826 in the training and validation sets, respectively, indicating that serum exosomal circ-PNN has the potential to become a new biomarker for CRC diagnosis in the future because of its high sensitivity. Recent research has contributed to a growing consensus that early diagnosis can prevent a substantial proportion of the suffering and mortality from CRC (37). Therefore, the diagnostic efficacy of circ-PNN for early stage of CRC was analyzed. Interestingly, the AUC was 0.854, indicated that circ-PNN also had good performance to distinguish early CRC cases from healthy controls.

Despite the research has proved that circRNAs plays an important role in oncogenesis and influences the proliferation, migration, and apoptosis in different cancer, the function of most circRNAs is still unclear. Bioinformatics analysis was performed to elucidate the functions and mechanisms of the circRNAs in CRC. We found some important functions or pathways that may explain the possible mechanisms of the increased circRNAs in CRC. For instance, overexpressed circRNAs are linked to chemokine signaling which can directly promote the progression of tumor by inducing the proliferation of cancer cells and preventing their apoptosis (38). To further understand the mechanisms of the circRNAs, we carefully searched and read the relevant studies, and found that circRNAs may function as miRNA sponges or potent competitive endogenous RNA (ceRNA) molecules to regulate gene expression (39–41). In this study, we predicted the potential miRNA targets of circ-PNN through miRanda and RNAhybrid database. We also found a tendency of negative correlation between circ-PNN and potential related miRNAs. The association of miRNAs with CRC indicated that circ-PNN may have a regulatory role in the development of CRC. For example, miR-1301 was significantly down-regulated in the serum of CRC patients and previous studies have reported that miR-1301 functions as a tumor suppressor that inhibits dissemination and metastasis of tumor cells (42). Of course, future studies are required to verify these bioinformatics analyses.

Nevertheless, the limitation of the present study should also be acknowledged that the sample size is still not large enough. Thus, further large-scale and multi-center samples are needed in the future to verify the diagnostic efficiency of serum exosomal circ-PNN before it could be adopted into clinical practice. Moreover, due to the short duration of our study and the limitations of patient prognosis information, we did not find a suitable data set to study the relation between serum exosomal circ-PNN and patient survival or treatment response. So, it is necessary to make effective strategies such as longer follow-up duration to investigate whether the expression level of serum exosomal circ-PNN can affect the prognosis of patients and whether the expression level changes after an effective treatment. Despite these limitations, we believe that the present evidences are enough to support our conclusion that serum exosomal circ-PNN may be a potential ideal biomarker for CRC. In addition, this study directly quantified the expression level of serum exosomal circ-PNN from fresh samples based on RT-qPCR assay, making it easy to implement in clinical practice.

In summary, based on our results, differentially expressed serum exosomal circRNAs were found in CRC through high-throughput sequencing of circRNA analysis, and were validated using RT-qPCR. ROC curve analysis was performed to demonstrate that circ-PNN has the potential to be a novel biomarker of high diagnostic accuracy in CRC. Our study also provides a solid theoretical foundation for exploring the role of these differentially expressed circRNAs in the pathogenesis of CRC, though the underlying mechanisms of circ-PNN in CRC tumorigenesis and development still need further investigation.

## Materials and Methods

### Clinical Samples

This study was a case–control study designed to examine serum exosomal circRNAs as potential biomarkers for CRC. A total of 221 serum samples (50 serum samples for sequencing, 88 assigned to the training set, 58 for the validation set, and 25 used to verify the expression correlation between circ-PNN and miRNA) were obtained from CRC patients, and 221 serum samples were obtained from healthy controls with no previous medical history of cancer. All samples were collected at The Second Hospital of Shandong University between 2017 and 2019. Patients with CRC were diagnosed by histopathology or histobiopsy, and samples were collected prior to any therapies. Tumor stage was defined according to the 2003 UICC tumor node metastasis (TNM) classification of CRC, and tumor grade was designated according to the WHO 2010 grading scheme. Written informed consent was obtained from all participants prior to serum samples collection. The study protocol was approved by the Clinical Research Ethics Committee of The Second Hospital of Shandong University.

### Serum and Preparation

To thoroughly remove cell debris, about 5 mL of venous blood obtained from each participant was centrifuged at 3,000 rpm for 10 min at 4°C, then 10,000 rpm for 10 min, within 2 h of collection. The supernatant (serum) was then stored at −80°C before use.

### High-Throughput Sequencing

For samples selected for sequencing, 50 serum samples of CRC patients were randomly divided into 5 groups, and the sera in each group were mixed evenly; 50 serum samples of healthy controls were also treated similarly. As a result, we had five sets of serum samples from CRC patients and five healthy control samples for sequencing. Transcriptome high-throughput sequencing and subsequent bioinformatics analyses were carried out by Cloud-Seq Biotech (Shanghai, China) as follows: First, the Ribo-Zero rRNA Removal Kit (Illumina, San Diego, CA, USA) and the CircRNA Enrichment Kit (Cloud-seq, USA) were applied to remove the rRNA and enrich the circRNAs. RNA libraries were constructed by using pretreated RNAs with TruSeq Stranded Total RNA Library Prep Kit (Illumina, San Diego, CA, USA), and the BioAnalyzer 2100 system (Agilent Technologies, Inc., Santa Clara, CA, USA) was used to control the quality and quantity of the libraries according to the manufacturer's instructions. After libraries were changed into single-stranded DNA molecules, the products were captured by Illumina Flow Cells, amplified *in situ* and sequenced for 150 cycles on Illumina HiSeq™ 4000 Sequencer (Illumina, San Diego, CA, USA) following the manufacturer's instructions.

### Sequencing Analysis of CircRNA

Paired-end reads were obtained from Illumina HiSeq 4000 sequencer, and the quality control were evaluated by Q30. Cutadapt software (v1.9.3) was used to trim the 3′adaptor and remove the poor-quality reads. The resulting high-quality trimmed reads were aligned to the reference genome/transcriptome by using STAR software, and circRNAs were explored and identified with DCC software. Circ2Trait database and circBase database were applied to annotate the identified circRNAs. Edger software was used to standardize the data and analyze the differentially expressed circRNAs. Significantly differentially expressed circRNAs (fold changes ≥ 2.0 and *P* < 0.05) between groups were identified for further use. GO and KEGG were applied to predict functions of these differentially expressed circRNAs.

### Analysis of CircRNA-miRNA-mRNA Network

The miRNA binding sites and target mRNAs were predicted by popular target prediction software: Targetscan and Miranda. For circ-PNN, we showed the top 5 predicted miRNAs and the five most likely downstream genes to every miRNA. Cytoscape software was applied to construct a circRNA-miRNA-mRNA network map based on data of circRNA with its miRNA-binding and predicted miRNA binding sites, to visualize the interactions between these molecules.

### Exosome Extraction

All the collected serum samples were centrifuged at 2,000 g for 30 min to thoroughly remove any cellular debris. Then the supernatant was combined with ExoQuick exosome precipitation solution (System Bio-sciences, CA, USA) according to the manufacturer's instructions. The samples were incubated and kept at 4°C for at least 30 min, after which they were centrifuged at 1,500 g for 30 min. The supernatant was discarded after centrifugation, and the pellets containing pure exosomes were re-suspended in 200 ul PBS and stored at −80°C prior to further use.

### RNA Extraction and Reverse Transcription Quantitative Polymerase Chain Reaction (RT-qPCR)

Total exosomal RNA was extracted from the exosome pellets using the miRNeasy Serum/Serum Kit (Qiagen, Hilden, Germany) according to the supplied protocol. Total RNA concentration was quantified by NanoDrop spectrophotometer (Thermo Fisher Scientific) at 260 nm. Subsequently, 1 ug RNA was incubated for 2 min at 42°C with 5 × g DNA Eraser Buffer and gDNA Eras, cDNA was synthesized from total RNA using Prime Script™ RT Reagent Kit (Takara, Dalian, Liaoning, China) at 37°C for 15 min and 85°C for 5 s according to the manufacturer's instructions to detect circRNA and mRNA. For the cDNA used to detect the expression of miRNA, 10 ul of reverse transcription (RT) reaction system containing 5 ul of 2 × miRNA Reaction Buffer Mix, 1.25 ul of miRNA Primescript RT Enzyme Mix and 3.75 ul RNA. The reactions were incubated at 37°C, 30 min, followed by 85°C for 5 s. The relative gene expression of RNA was determined using the CFX96 Real-Time PCR Detection System (Bio-Rad Laboratories, Hercules, CA, USA) as follows: 95°C for 30 s, followed by 40 cycles at 95°C for 5 s, and a pre-selected annealing temperature 60°C for 30 s was used as the PCR condition. Based on previous literature, the housekeeping gene β-actin was used to normalize the relative expression levels of circRNA and linear mRNA (43, 44), whereas U6 was utilized as the internal reference of miRNA (45, 46). The relative expressions of serum exosomal circRNAs were calculated by the comparative 2^−ΔΔCt^ method as follows: ΔCt = Ct _target gene_-Ct _internal reference_, and ΔΔCt = ΔCt _experimental group_−ΔCt _control group_ (47). Where Ct is the number of amplification cycles when the real-time fluorescence intensity of the reaction reached the set threshold. Three independent experiments were repeated in the qRT-PCR assay. The primers used for RT-qPCR target amplification are listed in Table S6.

### Western Blotting Analysis

In order to verify that the pellets isolated from the serum samples were truly exosomes, western blot analysis for the detection of CD9 and TSG101, was performed. Total protein was extracted by RIPA buffer (Thermo Scientific) containing protease inhibitor, and then we determined the concentration by using a bicinchoninic acid (BCA) protein assay kit (Pierce, Thermo Scientific). Then, 10 μg total protein were separated with sodium dodecyl sulfate polyacrylamide gel electrophoresis (SDS-PAGE) and then transferred to polyvinylidene difluoride (PVDF) membranes (Millipore, Hertfordshire, UK). After being blocked with 5% non-fat milk powder at room temperature for 1 h, the membrane was incubated with the following primary antibodies: anti-CD9 antibody (rabbit IgG) (13174S, CST, USA), and anti-TSG101 (mouse IgG) (Ab83, Abcam, UK). The following day, the membranes were treated with secondary antibodies: Goat anti-rabbit HRP secondary antibody (ZB-2301, ZSGB-BIO, China) and goat anti-mouse HRP secondary antibody (ZB-2305, ZSGB-BIO, China). The images were acquired using the Clarity Western ECL kit (Bio-Rad).

### Actinomycin D Treatment

Actinomycin D treatment reaction was performed according to the manufacturer's instructions. SW620 cells were seeded into six-well plates. After 60% confluency, cells were treated with 5 μg/ml Actinomycin D or 5 μg/ml DMSO as control and collected at indicated time points, the RNA expression levels of circ-PNN and other mRNAs were analyzed by RT-qPCR.

### Transmission Electron Microscopy (TEM)

We re-suspended the exosomes in 200 μL PBS, and then 20 μL aliquots were transferred to carbon-coated Cu grids (ProSciTech, Kirwan, QLD, Australia). The grids were allowed to stand for 10 min to absorb. Subsequently, samples were stained with 20 μL 3% glutaraldehyde fixative for 5 min and then dried. The grids were washed 10 times in DW, 2 min each time. Four percentage of uranium dioxyacetate was used for 10 min and 1% methyl cellulose for 5 min, after which they were allowed to dry. The morphologies of isolated exosomes were examined using a JEOL-1200EX TEM operated.

### Nanoparticle Tracking Analysis (NTA)

The size distribution and concentration of exosome pellets were determined using NTA. Briefly, 20 μg exosomes was diluted in 1 mL PBS and mixed well, the diameters of the exosomes and size distributions were measured by a ZETASIZER Nano series-Nano-ZS instrument (Malvern, UK). On the basis of Brownian motion and diffusion coefficient, particles were tracked and sized. All samples were subjected to similar detection thresholds. Filtered PBS were used as controls.

### Statistical Analysis

The non-parametric Mann–Whitney U-test was applied to compare the differences between healthy controls and CRC patients. The connection between two variables was determined by Spearman's correlation analysis. Scatter diagrams were plotted using GRAPHPAD PRISM 5 (San Diego, CA, USA). ROC curves and the area under the ROC curve (AUC) were performed using MEDCALC 15.2.2 (Med-Calc, Mariakerke, Belgium) to evaluate the diagnostic performance of the circRNA in CRC. We statistically analyzed the data with SPSS STATISTICS 22.0 (IBM, Chicago, IL, USA). A *P*-value < 0.05 was considered as statistically significant.

## Data Availability Statement

The datasets presented in this study can be found in online repositories. The names of the repository/repositories and accession number(s) can be found below: the NCBI Gene Expression Omnibus (GSE149200).

## Ethics Statement

The study protocol was approved by the Clinical Research Ethics Committee of The Second Hospital of Shandong University. Written informed consent was obtained from all participants.

## Author Contributions

YX and JL: performed the experiments, analyzed the data, and drafted the manuscript. JL, LD, and CW: initiated, organized, and supervised the study. LD, JL, and HB: critically revised the manuscript. YX, YZhan, and NL: collected urine samples. YC and YW: provided clinical information. PL, WD, YZhao, and NL: provided technical support. All authors read and approved the final version of the manuscript.

## Conflict of Interest

The authors declare that the research was conducted in the absence of any commercial or financial relationships that could be construed as a potential conflict of interest.
