# Supplementary material for: RNA-Seq Profiling of Serum Exosomal Circular RNAs Reveals Circ-PNN as a Potential Biomarker for Human Colorectal Cancer
Source: Front Oncol. 2020 Jun 18;10:982. doi: 10.3389/fonc.2020.00982 (PMC7314951; doi:10.3389/fonc.2020.00982)
Supplement: Supplementary file 1 [file Data_Sheet_1.pdf]

Supplementary Material

Table S1： Personal characteristics of CRC patients and healthy controls who participated in the sequencing [median (interquartile range)]

Variable	CRC (n=50)	Healthy controls (n=50)
**Age (years)**	64 (61-70)	63 (55-67)
**Sex**		
Male	29 (58%)	24 (48%)
Female	21 (42%)	26 (52%)
**Location**		
Colon	20 (40%)	
Rectum	30 (60%)	
**TNM stage**		
I-II	27 (54%)	
III-IV	23 (46%)	
**Tumor size**		
≤5 cm	34 (68%)	
>5 cm	16 (32%)	
**Lymph node metastasis**		
Negative	29 (58%)	
Positive	21(42%)	

Abbreviations: CRC, Colorectal cancer.

Table S2： The top 8 significantly upregulated circRNAs

（Expressed in at least two samples）

CircRNA ID	CircBase ID	Gene symbol	logFC	P-value
chr3:138289160-138291774-	hsa_circ_0004524	CEP70	9.018253	0.028383
chr2:106774514-106782539-	hsa_circ_0001060	UXS1	8.661636	0.032282
chr11:73418465-73429763-	hsa_circ_0000339	RAB6A	8.568319	0.03344
chr15:76584772-76588078-	hsa_circ_0003620	ETFA	8.528082	0.033922
chr20:50245424-50245575-	NA	ATP9A	8.518475	0.034047
chr17:80521230-80526077+	hsa_circ_0000816	FOXK2	8.51134	0.034138
chr7:102962379-102963241-	hsa_circ_0081751	DNAJC2	8.454866	0.034847
chr14:39648295-39648666+	hsa_circ_0101802	PNN	8.4546	0.034867

NA, not available.

Table S3. Characteristics of study participants in the training and validation sets [median (interquartile range)]

Variable	Training set (n=176)	Validation set (n=116)
**Healthy Control (number)**	88	58
**Age (years)**	53 (48-58)	55 (48-58)
**Sex**		
Male	49 (55.68%)	28 (48.28%)
Female	39 (44.32%)	30 (51.72%)
**CRC (number)**	88	58
**Age (years)**	63 (54-69)	65 (59-70)
**Sex**		
Male	57 (64.77%)	39 (67.24%)
Female	31 (35.23%)	19 (32.76%)
**Location**		
Colon	33(37.50%)	20(34.48%)
Rectum	55(62.50%)	38(65.52%)
**TNM stage**		
I-II	38 (43.18%)	36 (62.07%)
III-IV	50 (56.82%)	22 (37.93%)
**Tumor size**		
≤5cm	64 (72.73%)	38 (65.52%)
＞5cm	24 (27.27%)	20 (34.48%)

**Lymph node metastasis**		
Negative	44(50.00%)	36 (62.07%)
Positive	44 (50.00%)	22 (37.93%)

Abbreviations: CRC, colorectal cancer

Table S4. Correlations between concentrations of circ-PNN and clinicopathological characteristics of CRC patients in the validation set [median (interquartile range)]

Parameters	Total cases	circ-PNN	*p*
**Age (years)**			0.15
＜65	31	2.04 (1.51-3.32)	
≥65	27	2.95 (1.78-4.86)	
**Sex**			0.29
Male	39	2.95 (1.51-4.89)	
Female	19	2.55 (1.74-3.20)	
**Location**			0.20
Colon	20	1.95 (1.30-4.14)	
Rectum	38	2.83 (1.75-4.52)	
**TNM stage**			0.05
I-II	35	3.13 (1.80-4.91)	
III-IV	23	2.08 (1.38-3.188)	
**Tumor size**			0.61
≤5 cm	38	2.67(1.56-4.87)	
>5 cm	20	2.66(1.52-4.31)	
**Lymph node metastasis**			0.21

Negative	36	3.02 (1.75-4.92)
Positive	22	2.42 (1.48-3.20)

Abbreviations: CRC, Colorectal cancer.

Table S5: Personal characteristics of participants to analyze the relationship between mirna and circRNA expression level. [median (interquartile range)]

Variable	CRC(n=25)	Healthy controls (n=25)
**Age (years)**	63 (46-75)	54 (45-65)
**Sex**		
Male	16 (64%)	9 (26%)
Female	9 (26%)	16 (64%)
**Location**		
Colon	7 (28%)	
Rectum	18 (72%)	
**TNM stage**		
I-II	13 (52%)	
III-IV	12 (48%)	
**Tumor size**		
≤5 cm	17 (68%)	
>5 cm	8 (32%)	
**Lymph node metastasis**		
Negative	17 (68%)	
Positive	8 (32%)	

Abbreviations: CRC, Colorectal cancer.

Table S6. Primer sequences

Gene	Primer
**chr3:138289160-138291774-**	Forward primer 5'-GGAGCAGAAAACTTTACAGTAAC-3'<br>Reverse primer 5'-CCATGCATCATCAATAGCACA-3'
**chr2:106774514-106782539-**	Forward primer 5'- CGGAAAAGAATTTTGCTGTTTG-3'<br>Reverse primer 5'- CTTTACTGGTGGGTATTTCT-3'
**chr11:73418465-73429763-**	Forward primer 5'-GATACAATGTAAAGCAGGTACG-3'<br>Reverse primer 5'-CATGATGATAACATCACTTCCTC-3'
**chr15:76584772-76588078-**	Forward primer 5'- TGCCTTCGGAAAGGCCTCAT-3'<br>Reverse primer 5'- GTTCCAGCTACTAAGCAGGAC-3'
**chr17:80521230-80526077+**	Forward primer 5'-GAACCATCAGCGCTGCAAAC-3'<br>Reverse primer 5'-GAACCTGAATGTGCACCTTCG-3'
**chr7:102962379-102963241-**	Forward primer 5'- GAAGCTGCTCGGTTAGCTAAG-3'<br>Reverse primer 5'-TCTCCTCTCATCACGACTTGC-3'
**chr14:39648295-39648666+**	Forward primer 5'-GCTTGCGCAGCTGCAAGAAG-3'<br>Reverse primer 5'- TGGCGCCGCTTTTGCGTTCA-3'
**PNN**	Forward primer 5'-CAATCCCAGTCCCAACCAGT-3'<br>Reverse primer 5'-TAACTTGGGGTGTTGGCTGT-3'
**β-actin**	Forward primer 5'-CATGTACGTTGCTATCCAGGC-3'<br>Reverse primer 5'-CTCCTTAATGTCACGCACGAT-3'
**Hsa-miR-6833-3p**	Forward primer 5'-TTTCTCTCTCCACTTCCTCAG-3'<br>Reverse primer: part of the Mir-X™ miRNA First-Strand Synthesis.
**Hsa-let-7i-3p**	Forward primer 5'- CCAGCTACTGCCTTGCTAAA-3'<br>Reverse primer: part of the Mir-X™ miRNA First-Strand Synthesis.
**Hsa-miR-1301-3p**	Forward primer 5'-CTGCCTGGGAGTGACTTCAAA-3'<br>Reverse primer: part of the Mir-X™ miRNA First-Strand Synthesis.

**U6**	Forward primer 5’-GGAACGATACAGAGAAGATTAGC-3’<br>Reverse primer 5’-TGGAACGCTTCACGAATTTGCG-3’
